# Individualized Treatment Strategy for Cutaneous Melanoma: Where Are We Now and Where Are We Going?

**DOI:** 10.3389/fonc.2021.775100

**Published:** 2021-11-04

**Authors:** Huihua Zeng, Fen Liu, Hairong Zhou, Changchun Zeng

**Affiliations:** ^1^ Department of General Medicine, Shenzhen Longhua District Central Hospital, Shenzhen, China; ^2^ Department of Chinese Medicine, Shenzhen Longhua District Central Hospital, Shenzhen, China; ^3^ Department of Medical Laboratory, Shenzhen Longhua District Central Hospital, Guangdong Medical University, Shenzhen, China

**Keywords:** MAPK, melanoma, targeted therapy, immunotherapy, resistance

## Abstract

In the past several decades, innovative research in cancer biology and immunology has contributed to novel therapeutics, such as targeted therapy and immunotherapy, which have transformed the management of patients with melanoma. Despite the remarkable therapeutic outcomes of targeted treatments targeting MAPK signaling and immunotherapy that suppresses immune checkpoints, some individuals acquire therapeutic resistance and disease recurrence. This review summarizes the current understanding of melanoma genetic variations and discusses individualized melanoma therapy options, particularly for advanced or metastatic melanoma, as well as potential drug resistance mechanisms. A deeper understanding of individualized treatment will assist in improving clinical outcomes for patients with cutaneous melanoma.

## Introduction

Currently, melanoma is the most common type of skin cancer. Despite accounting for approximately 1% of skin malignancies, melanoma is responsible for most skin cancer-related mortality. GLOBOCAN 2020 estimates that 324,635 new skin melanoma cases and 57,043 new melanoma of skin deaths occurred in 2020 (1). In 2021, approximately 101,280 new skin melanoma cases are predicted in the United States. The incidence of skin melanoma continues to rise, but it has begun to decline in recent birth cohorts. From 2009 to 2013, the melanoma mortality rate held steady but then dropped by 5.7% yearly for the following five years. Recently, metastatic melanoma death rates have been steadily declining, possibly owing to advances in therapy. The 5-year survival rate for early-stage melanoma is 99%, but just 27% for metastatic melanoma. When melanoma is detected early, it is feasible to treat it surgically, resulting in increased survival rates. However, therapeutic interventions become restricted if the melanoma has progressed to distant organs (2).

Primary melanoma is linked to a variety of risk factors, including male sex, old age (>60 years), phenotypic predisposition (atypical mole or dysplastic nevus pattern, increased mole count, sun-phenotype or tendency to sunburn, red hair-blue eyes or Fitzpatrick skin type I or pheomelanin predominant phenotype), personal medical history or comorbidities (multiple or blistering sunburns, actinic keratosis or non-melanoma skin cancer, childhood cancer, solid organ transplantation, hematopoietic cell transplantation, human immunodeficiency virus or acquired immunodeficiency syndrome, xeroderma pigmentosum), environmental factors (tanning bed use, residence in sunnier climate or latitude nearer to equator, intermittent, intense sun exposure, chronic sun exposure), and genetic predisposition (family history of cutaneous melanoma, pancreatic, renal and/or breast cancer, astrocytoma, uveal melanoma, and/or mesothelioma, germline mutation, including CDKN2A, CDK4, MC1R, BRCA2, BAP1, TERT, MITF, and PTEN) (3–7).

BRAF, MEK, NRAS, HRAS, KRAS, c-KIT, c-Met, VEGFR, PTEN, and PIK3CA mutations are common in melanoma. Activation of Ras/Raf/MAPK, PI3K/AKT/mTOR, and JAK/STAT signaling pathways leads to malignant phenotypes and tumorigenesis (8, 9). Advances in the study of biological functions and molecular mechanisms may provide potential therapeutic targets for patients with melanoma. The revolutionary discoveries of targeted therapy and immunotherapy, two highly effective treatment strategies, profoundly altered the standard of practice for melanoma, leading to a renewed hope of preventing the disease (10). Unfortunately, not all patients respond to targeted or immunotherapy. Moreover, most patients eventually develop drug resistance and lack treatment options. Therefore, research into resistance mechanisms and novel treatment alternatives remains critical. Here, we present an overview of treatment options and future perspectives in melanoma.

## Genetic Alterations in Melanoma

Specific genetic alterations regulate the onset and development of melanoma. The common deleterious mutations closely associated with melanoma were identified (**Table 1**). The functional mutation burden of BRAF, CDKN2A, MAP2K1, NRAS, PTEN, TP53, PPP6C, RAC1, SNX31, TACC1 and STK19 was statistically significant. Deletions of PTEN and CDKN2A and gains of CCND1, MITF, and TERT, were identified in somatic copy-number alterations profiles. 83% (100/121) of melanoma cases had highly recurrent mutations in NRAS (27/121) or BRAF (n=73/121) that were mutually exclusive. Highly recurrent mutations in the BRAF V600 codon, which accounts for 35-50% of melanoma, and the Q61 codon, which accounts for 10-25% of melanoma, contribute to the evolution of highly selective kinase inhibitors for the MAPK pathway (9). 13 significantly mutated genes (SMGs), including BRAF, NRAS, CDKN2A, TP53, PTEN, RAC1, MAP2K1, PPP6C, and ARID2 were identified using functional mutation burden and loss-of-function tests. UV-induced highly recurrent alterations were identified in SMGs, including IDH1 (6.2%) and RAC1 (6.9%). In addition, abnormal activation of RAS/MAPK/AKT, cell cycle, and apoptosis pathway occurred in 91%, 69%, and 19% of samples, respectively. According to the pattern of SMGs, cutaneous melanomas can be classified into four genomic subgroups, including mutant BRAF, mutant RAS, mutant NF1, and Triple-WT (wild-type) (8).

**Table 1:** Molecular alterations in melanoma.

Gene	Mutation	Locus	Freq (%)	Pathway	Reference
CCND1	Mut; amp	11q13	5	Cell-cycle pathway	(9, 11, 12)
CDK4	Mut; amp	12q14	5	Cell-cycle pathway	(9, 11, 12)
CDKN2A	Mut; del; hm	9p21	29	Cell-cycle pathway	(9, 11, 12)
CDKN2B	Mut; del; hm	9p21	18	Cell-cycle pathway	(9, 11, 12)
RB1	Mut	13q14.2	5	Cell-cycle pathway	(9, 11, 12)
TP53	Mut	17p13.1	14	DNA damage response and cell death pathways	(9, 11, 12)
ARID2	Mut	12q12	13	Epigenetics	(9, 11, 12)
EZH2	Mut	7q35-q36	7	Epigenetics	(9, 11, 12)
IDH1	Mut	2q33.3	5	Epigenetics	(9, 11, 12)
BRAF	Mut; amp; fusion	7q34	49	MAPK pathway	(9, 11, 12)
GNA11	Mut	19p13.3	3	MAPK pathway	(9, 11, 12)
GNAQ	Mut	9q21	2.2	MAPK pathway	(9, 11, 12)
HRAS	Mut	11p15.5	1.3	MAPK pathway	(9, 11, 12)
KDR	Mut; amp	4q11-q12	13	MAPK pathway	(9, 11, 12)
KIT	Mut; amp	4q12	6	MAPK pathway	(9, 11, 12)
KRAS	Mut	12p12.1	2.2	MAPK pathway	(9, 11, 12)
MAP2K1	Mut; amp	15q22.31	6	MAPK pathway	(9, 11, 12)
MTOR	Mut	1p36.2	9	MAPK pathway	(9, 11, 12)
NF1	Mut	17q11.2	13	MAPK pathway	(9, 11, 12)
NRAS	Mut; amp	1p13.2	24	MAPK pathway	(9, 11, 12)
PDGFRA	Mut; amp	4q12	9	MAPK pathway	(9, 11, 12)
RAC1	Mut; amp	7p22	7	MAPK pathway	(9, 11, 12)
AKT3	Mut; amp; fusion	1q44	5	PI3K/Akt/mTOR Pathway	(9, 11, 12)
PIK3CA	Mut	3q26.3	4	PI3K/Akt/mTOR Pathway	(9, 11, 12)
PTEN	Mut; del	10q23.3	13	PI3K/Akt/mTOR Pathway	(9, 11, 12)
TSC1	Mut; amp	9q34	4	PI3K/Akt/mTOR Pathway	(9, 11, 12)
TSC2	Mut	16p13.3	6	PI3K/Akt/mTOR Pathway	(9, 11, 12)
TERT	Promoter mut	5p15.33	7	Telomerase pathway	(9, 11, 12)

The frequency was obtained using data from a combined study in The Cancer Genome Atlas (TCGA) database (https://www.cbioportal.org/). Skin Cutaneous Melanoma (TCGA, Firehose Legacy, N=479 samples), Skin Cutaneous Melanoma (TCGA, PanCancer Atlas), Skin Cutaneous Melanoma (Broad, Cell 2012) (9), Skin Cutaneous Melanoma (Yale, Nat Genet 2012) (11), and Skin Cutaneous Melanoma (Broad, Cancer Discov 2014) (12) were all included in the combined study. MUT, mutation; Amp, amplification; Del, deletion; Hm, hypermethylation; Freq, Frequency.

**Figure 1:**
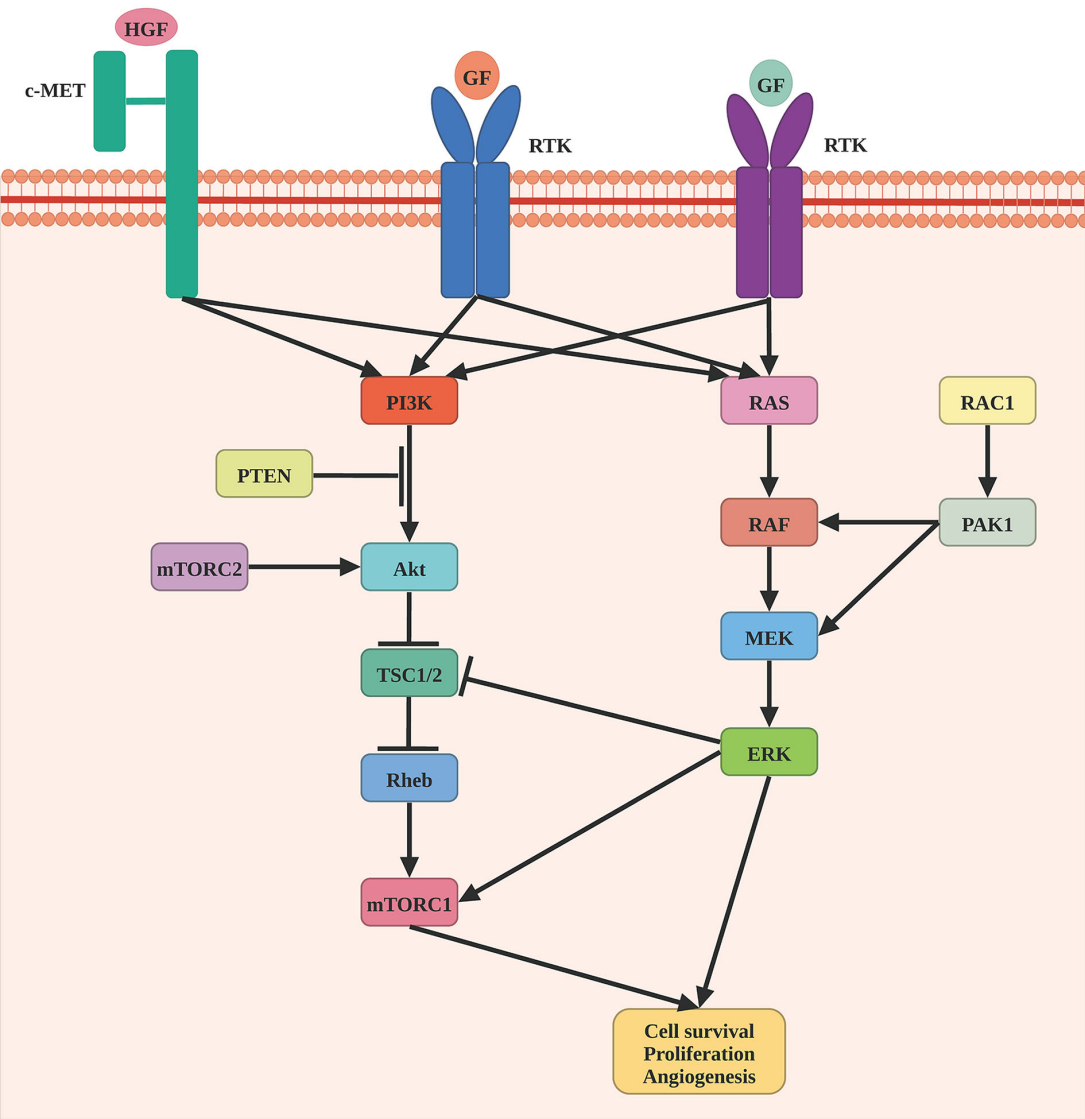
Schematic representation of MAPK pathway in melanomagenesis. Oncogenic mutations in BRAF are the most common genomic alterations in melanoma, followed by a mutation in NRAF, NF1, and RAC1.

Braf is a component of the RAS/MAPK signaling pathway, which regulates a variety of critical cellular processes, such as cell proliferation, differentiation, migration, and apoptosis (**Figure 1**). BRAF somatic mutations were identified in 52.2% of 318 patients. V600E (n = 124), V600K (n = 18), and V600R (n = 3) were among the 145 patients that targeted the V600 amino acid residue. The K601 residue was the target of the second most common BRAF mutation (n = 5). BRAF V600 and K601 hot-spot mutations were negatively associated with hot-spot NRAS mutations. BRAF non-hot-spot mutations, on the other hand, frequently co-occurred with RAS hot-spot and NF1 mutations. The Nras protein, a GTPase, converts GTP to GDP. NRAS hot-spot mutations result in decreased GTPase activity, which leads to enhanced GTP-bound Nras and activation of downstream pathways, such as RAF/MEK, PI3K/AKT, and RALGEF/RALA/RALB pathway (13, 14). NRAS somatic mutations were identified in 28.4% of 88 patients. Among them, 97.7% (n=88) of these patients harbored NRAS hot-spot mutations, including Q61R (n=33), Q61K (n=28), Q61L (n=11), Q61H (n=4), 61_62QE > HK (n=1), and G13R/D (n=3). Other RAS family members, such as hot-spot HRAS (G13D, G13S, and Q61K) and KRAS (G12D, G12R, and Q61R), were shown to have mutations that were mutually exclusive with NRAS and BRAF V600 and K601 mutations. Patients carrying BRAF mutations were considerably younger than those carrying other mutations, while those with NF1 mutations tended to be older. NF1 mutation was detected in 14% of cases, most of which are loss-of-function events. Nf1 is a Ras pathway negative regulator that may stimulate RAS GTPase activity, causing cell growth and malignancy. Besides, the MAPK signaling pathway can be stimulated by loss-of-function mutation of NF1. Moreover, the NF1 subtype exhibited a significantly high mutation burden (8, 15, 16).

BRAF, MITF, and CD274 amplifications were found at substantial frequencies in patients with BRAF mutations, while NRAS amplifications occurred in patients with NRAS mutations. PTEN deletions and mutations were more prevalent in the BRAF-mutant subgroup, while mRNA overexpression and amplification of AKT3 were considerably more prevalent in RAS, NF1, and Triple-WT subtypes, which may serve as new biomarkers to justify the use of MEK and PI3K/AKT/mTOR pathway inhibitors in these subtypes. Moreover, a UV signature was observed in 90.7% (136/151) of BRAF, 93.5% (86/92) of RAS, and 92.9% (26/28) of NF1 subtypes but only 30% (14/46) of Triple-WT. Although the Triple-WT lacked a UV signature, this subtype exhibited high copy-number alterations and complex rearrangements. KIT, PDGFRA, KDR, CDK4 and CCND1, MDM2, and TERT amplifications were frequently observed in Triple-WT melanomas. Besides, TERT promoter mutations were identified in 75.0% (n=52) of BRAF, 71.9% (n=32) of RAS, and 83.3% (n=12) of NF1 subtypes but in only 6.7% (n=15) of Triple-WT (8).

CDKN2A is notable for encoding two vastly distinct proteins: p16INK4a and p14ARF. p16INK4a functions as a cyclin-dependent kinase inhibitor in the RB1 cell cycle pathway, while p14ARF interacts with the p53-stabilizing protein MDM2 in the p53 pathway. Alterations in CDKN2A in melanoma mainly target p16INK4a or affect both p16INK4a and p14ARF. Additionally, there is a subgroup of less frequent somatic and germline INK4a/ARF alterations that impact p14ARF but not p16INK4a (17). Bi-allelic loss of CDKN2A is correlated with aggressive initiation of melanoma. BRN2 is transcriptionally triggered by the loss of p16INK4A through direct binding to E2F1 due to CDK4/6-mediated hyperphosphorylation of RB1 (18).

RAC1 encodes a GTPase that is a member of the Ras superfamily of small GTP-binding proteins. Numerous protein kinases act as RAC1 effectors, providing an avenue for pharmacological suppression. The RAC1 P29S mutation is a frequent UV-signature mutation that is found in 9.2% of sun-exposed melanomas. The RAC1 P29S mutation was identified in both primary (9.2%) and metastatic (8.6%) melanoma, indicating that it is present early in carcinogenesis (11). In melanocytes, RAC1P29S stimulates PAK, AKT, and the SRF/MRTF transcription pathway and promotes a shift from melanocytic to mesenchymal cells through SRF/MRTF and PAK. Besides, RAC1 P29S in conjunction with BRAF mutations or NF1 deletions stimulates tumorigenesis. RAC1 P29S confers resistance to BRAF inhibitors through the SRF/MRTF signaling pathway, indicating that targeted SRF/MRTF therapy may overcome BRAF inhibitor resistance in melanoma patients with RAC1P29S mutations. Moreover, RAC1 P29S exhibited enhanced binding to PAK1, MLK3, and the WAVE complex. MLK3 can recruit a BRAF-RAF1 complex, implying that it may serve as a link between the RAC1 and MAP kinase cascades. Therebefore, RAC1 may be a therapeutic target in melanoma (11, 19).

## Therapy Targeting BRAF and MEK

**Figure 2:**
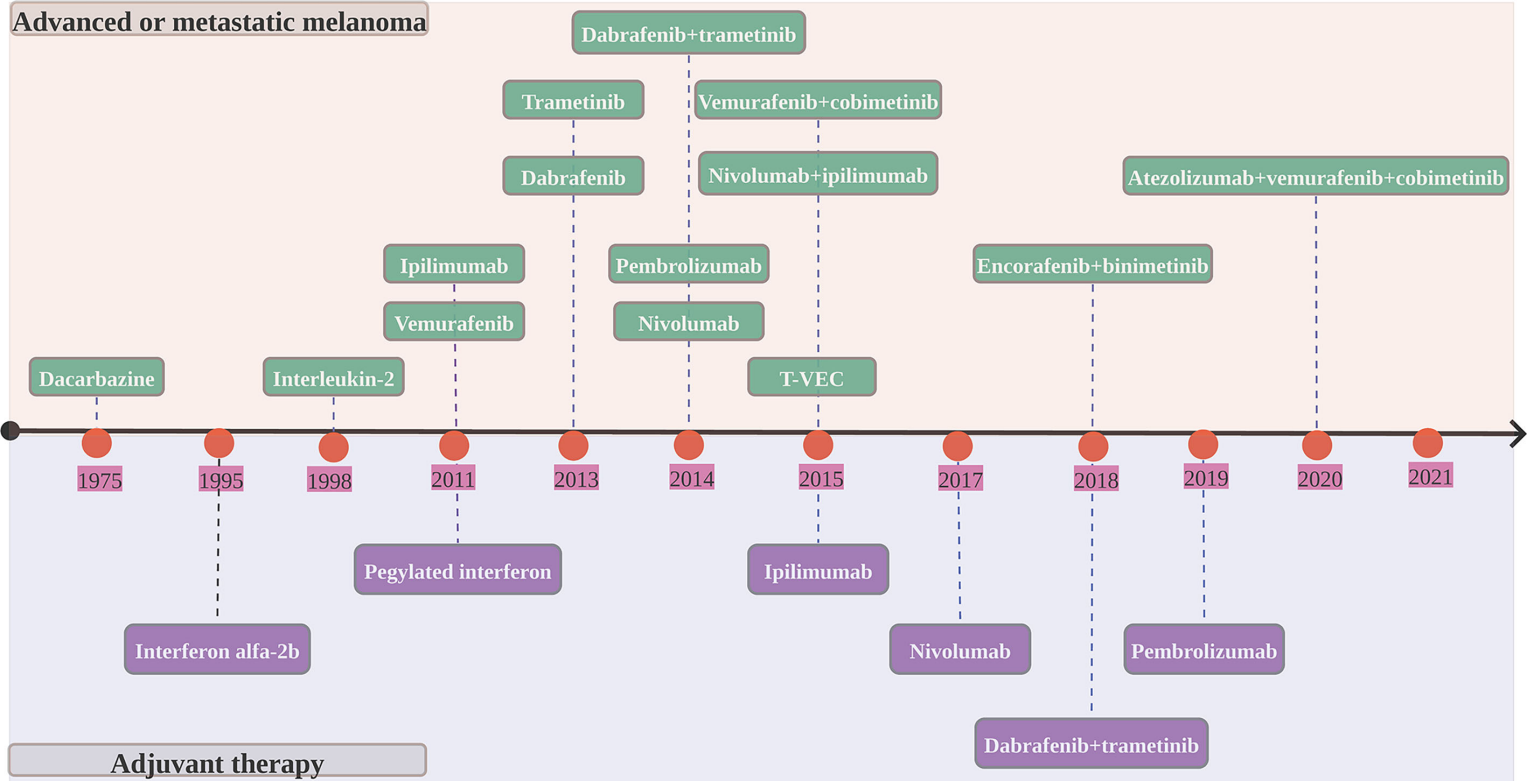
Timeline of FDA-approved melanoma therapies. T-VEC, talimogene laherparepvec.

In 2011, the Food and Drug Administration (FDA) approved vemurafenib as the first BRAF targeted therapy for unresectable or metastatic BRAF V600E-mutated melanoma based on an overall survival rate of 84%and a 63% decrease in the risk of mortality from the BRIM 3 trial (20). Despite its clinical significance, vemurafenib monotherapy rapidly develops resistance *via* reactivating mitogen-activated protein (MAP) kinase. The combination of BRAF and MEK inhibition that tackle this resistance mechanism has become the standard of treatment for melanoma patients. Targeted therapy with BRAF inhibitor in conjunction with MEK inhibitor has long-term benefits, but acquired resistance may restrict long-term disease management (**Table 2** and **Figure 2**).

**Table 2:** Melanoma treatment options (targeted and immune therapies).

Drugs	Drug targets	Mechanism	Indications
Ipilimumab	CTLA-4	Anti-CTLA-4 inhibitor	Unresectable or metastatic melanoma (NCT00094653; 2011)
Vemurafenib	BRAF V600E, CRAF, ARAF, wild-type BRAF, SRMS, ACK1, MAP4K5 and FGR	BRAF inhibitor	Unresectable or metastatic melanoma with the BRAF V600E mutation (NCT01006980; 2011)
Trametinib	MEK1, MEK2	MEK inhibitor	Unresectable or metastatic melanoma with BRAF V600E or V600K mutation (NCT01245062; 2013)
Dabrafenib	BRAF V600, wild-type BRAF, CRAF kinases, SIK1, NEK11, and LIMK1	BRAF inhibitor	Unresectable or metastatic melanoma with BRAF V600E mutation (NCT01227889; 2013)
Dabrafenib+trametinib	BRAF V600, wild-type BRAF, CRAF kinases, SIK1, NEK11, and LIMK1; MEK1, MEK2	BRAF inhibitor+MEK inhibitor	Unresectable or metastatic melanoma with a BRAF V600E or V600K mutation (NCT01584648; 2014)
Pembrolizumab	PD-1	Anti-PD-1 inhibitor	Unresectable or metastatic melanoma with disease progression following treatment with ipilimumab and, in BRAF V600 mutation–positive patients after treatment with a BRAF inhibitor (NCT01295827; 2014)
Nivolumab	PD-1	Anti-PD-1 inhibitor	Unresectable or metastatic melanoma with disease progression following treatment with ipilimumab and, in BRAF V600 mutation–positive patients after treatment with a BRAF inhibitor (NCT01721772; 2014)
Vemurafenib+cobimetinib	BRAF V600E, CRAF, ARAF, wild-type BRAF, SRMS, ACK1, MAP4K5 and FGR; MEK1, MEK2	BRAF inhibitor+MEK inhibitor	Unresectable or metastatic melanoma with BRAF V600E or V600K mutation (NCT01689519; 2015)
Nivolumab+ipilimumab	PD-1; CTLA-4	Anti-PD-1 inhibitor+Anti-CTLA-4 inhibitor	BRAF V600 wild-type, unresectable or metastatic melanoma (NCT01844505; 2015)
Ipilimumab	CTLA-4	Anti-CTLA-4 inhibitor	Adjuvant treatment of patients with cutaneous melanoma with pathologic involvement of regional lymph nodes of more than 1 mm who have undergone complete resection, including total lymphadenectomy (NCT00636168; 2015)
Nivolumab	PD-1	Anti-PD-1 inhibitor	Adjuvant treatment of patients with melanoma with involvement of lymph nodes or in patients with metastatic disease who have undergone complete resection (NCT02388906; 2017)
Encorafenib+binimetinib	BRAF V600, JNK1, JNK2, JNK3, LIMK1, LIMK2, MEK4, and STK36; MEK1, MEK2	BRAF inhibitor+MEK inhibitor	Unresectable or metastatic melanoma with a BRAF V600E or V600K mutation (NCT01909453; 2018)
Dabrafenib+trametinib	BRAF V600, wild-type BRAF, CRAF kinases, SIK1, NEK11, and LIMK1; MEK1, MEK2	BRAF inhibitor+MEK inhibitor	Adjuvant treatment of patients with melanoma with BRAF V600E or V600K mutations (NCT01682083; 2018)
Pembrolizumab	PD-1	Anti-PD-1 inhibitor	Adjuvant treatment of patients with melanoma with involvement of lymph node(s) following complete resection (NCT02362594; 2019)
Atezolizumab+vemurafenib+cobimetinib	PD-L1; BRAF V600E, CRAF, ARAF, wild-type BRAF, SRMS, ACK1, MAP4K5 and FGR; MEK1, MEK2	Anti-PD-L1 inhibitor+BRAF inhibitor+MEK inhibitor	BRAF V600 mutation-positive unresectable or metastatic melanoma (NCT02908672; 2020)

Based on the COMBI-v trial (dabrafenib plus trametinib *vs*. vemurafenib) (21) and COMBI-d trial (dabrafenib plus trametinib to dabrafenib) (22), the 5-year progression-free survival rates and overall rates for unresectable or metastatic melanoma with a BRAF V600 mutation treated with dabrafenib plus trametinib as first-line therapy were 19% and 34%, respectively (23). The coBRIM trial exhibited that first-line vemurafenib combined with cobimetinib improved progression-free and overall survival compared with vemurafenib in patients with BRAF V600-mutated advanced melanoma. The median overall survival of cobimetinib and vemurafenib (22.5 months) was superior to that of vemurafenib (17.4 months) after a median follow-up of 21.2 months. In addition, cobimetinib combined with vemurafenib had a median progression-free survival of 12.6 months versus 7.2 months for vemurafenib (24–26). In the COLUMBUS trial, the median overall survival of patients with BRAF V600-mutant melanoma was 33.6 months with COMBO450 (encorafenib plus binimetinib), 23.5 months with ENCO300 (encorafenib), and 16.9 months with VEM (vemurafenib). COMBO450 reduced the risk of mortality by 39% when compared to VEM. The median progression-free survival was 14.9 months with COMBO450, 9.6 months with ENCO300, and 7.3 months with VEM (27, 28). Overall, treatment with combined BRAF and MEK inhibition, such as dabrafenib plus trametinib, vemurafenib plus cobimetinib, and encorafenib plus binimetinib seems to provide longer progression-free and overall survival compared to treatment with BRAF inhibitor monotherapy.

The five-year analysis of adjuvant dabrafenib with trametinib in stage III melanoma with BRAF V600 mutations was presented in the COMBI-AD trial. With dabrafenib plus trametinib, 52% of patients survived without recurrence, compared to 36% with placebo. Moreover, 65% of patients survived without distant metastasis with dabrafenib plus trametinib, compared to 54% with placebo (29, 30). The NeoCombi study showed that among resectable, stage III, BARF V600 mutation melanoma, neoadjuvant dabrafenib plus trametinib therapy contributed to a RECIST response in 86% of patients and a complete pathological response with no progression in 49% of patients during neoadjuvant therapy (31).

Several hotspots of the KIT gene are oncogenic, and these hotspots exhibit variable responsiveness to KIT inhibitors, such as imatinib, sunitinib, nilotinib. Patients with metastatic melanoma harboring KIT mutation or amplification who received imatinib had a median progression-free survival of 3.5 months and a 6-month PFS rate of 36.6% (32). The appearance of mutations in KIT exons 11 and 13, such as W557, V559, L576P, K642E exhibited a high degree of sensitivity to KIT inhibition. KIT exon 17 mutations such as D816H seem to be less sensitive to KIT inhibitors. KIT amplification or NRAS mutations seem to be less sensitive or insensitive to KIT inhibitors (33–35).

The NEMO trial exhibited that advanced NRAS-mutant melanoma patients receiving binimetinib had a median progression-free survival of 28 months while advanced NRAS-mutant melanoma patients receiving dacarbazine had a median progression-free survival of 15 months after a median follow-up of 1.7 months (36).

## Potential Mechanisms of Resistance to Targeted Therapy

Most melanomas had oncogenic alterations such as RAC1 and AKT3 that stimulate the MAPK and PI3K pathways, reducing sensitivity to MAPK inhibitors (37). SEC61-dependent ER translocation of the MAPK pathway was triggered by BRAF and MEK inhibitors through GRP78 and KSR2. ER translocation inhibition inhibited ERK reactivation and autophagy. ERK reactivation contributed to the phosphorylation of AFT4, which triggered cytoprotective autophagy. In patients resistant to BRAF and MEK inhibitors, GRP78 upregulation and ATF4 phosphorylation were found, suggesting that ER translocation of the MAPK pathway causes treatment resistance in BRAF-mutant melanoma (38). PAKs phosphorylated CRAF and MEK to stimulate ERK in cells that are resistant to BRAF inhibitor. In cells resistant to combined BRAF and MEK inhibition, PAKs modulate the phosphorylation of JNK and β-catenin and the activation of the mTOR pathway, supporting that PAK signaling is responsible for acquired resistance to MAPK inhibitors in BRAF-mutant melanoma (39). Belvarafenib is a selective RAF dimer (type II) inhibitor with clinical efficacy in BRAF and NRAS-mutant melanomas. ARAF mutants develop resistance to belvarafenib in a kinase- and dimer-dependent manner. Combined RAF and MEK inhibition may postpone ARAF-driven resistance (40). The RAC1 P29S mutation works along with BRAF to promote melanoma initiation and cause resistance to BRAF inhibitors. In melanoma, the transcription factor complex SRF/MRTF is a key RAC1 effector, which has generated a therapeutic resistance by inducing a mesenchymal-like state (19). SPRED1 inactivation is common in BRAF-mutated melanoma. SPRED1 deficiency enhanced melanoma cell proliferation under mutant BRAF inhibition *via* the reactivation of MAPK activity. SPRED1 loss has been found in patients whose melanoma had developed resistance to MAPK-targeted treatment (41). Combined MEK and CDK4/6 inhibition is a promising therapeutic option in mutant BRAF and NRAS melanoma. The ribosomal S6 protein phosphorylation promotes the activation of the mTOR signaling pathway and leads to acquired drug resistance, supporting that targeting mTORC1/2 may overcome MEK plus CDK4/6 inhibition resistance (42). In patients with NRAS-mutant melanoma, combined MEK and CDK4/6 inhibitors have demonstrated encouraging therapeutic results. However, an acquired PIK3CA E545K mutation can depend on the S6K1 signaling pathway for drug resistance. S6K1 inhibition may resensitize NRAS-mutant melanoma carrying PIK3CA E545K to combined MEK and CDK4/6 inhibition (43).

Due to primary and secondary resistance, MEK inhibitors have shown limited effectiveness in patients with NRAS-mutated melanoma. Two patients with NRAS-mutant metastatic melanoma had a long-term response to intermittent binimetinib (MEK inhibitor). Intermittent administration may help overcome resistance, which is associated with the fact that it may lead to a fitness deficit for drug-resistant cells, enhance immunogenicity, enhance expression of immunomodulatory molecules, reduce immunosuppressive factors, and induce apoptosis and cell cycle arrest (44).

Exploratory biomarker analyses from the COMBI-AD study (NCT01682083) exhibited that the presence of MAP2K1/MEK1 mutations, BRAF amplification, or MAPK pathway gene (NRAS, KRAS, HRAS, MAP2K1/MEK1, MAP2K2/MEK2, MAPK1, NF1, MAPK7, or MAPK3) mutations at baseline did not affect clinical benefit. A significant increase in relapse-free survival was observed in patients with a high IFNγ gene expression signature in the dabrafenib plus trametinib group and placebo group. Tumor mutational burden (TMB) was independently associated with a higher likelihood of relapse-free survival in the placebo group. However, TMB was not associated with relapse-free survival in patients treated with dabrafenib plus trametinib. Besides, patients with a lower TMB benefited more from dabrafenib plus trametinib adjuvant treatment. Specifically, the subgroup with low TMB and high IFN gene expression signature benefited the most from targeted treatment (45).

Baseline genetic characteristics of BRAF V600-mutated metastatic melanoma patients treated with cobimetinib plus vemurafenib or vemurafenib alone showed that MITF and TP53 alterations were more frequent in patients who progressed rapidly, while NF1 alterations were more frequent in patients who obtained a complete response, which is consistent with the view that melanomas that lack NF1 expression are more dependent on the MAPK signaling pathway and are more sensitive to MAPK pathway inhibitors. Additionally, immune-related gene signatures such as CD8+ effector T cells, cytolytic T cells, antigen-presenting cells, and natural killer cells gene signatures were associated with complete response, while keratinization-related gene expression was associated with rapid progression (46).

## Immune Checkpoint Inhibitors

**Figure 3:**
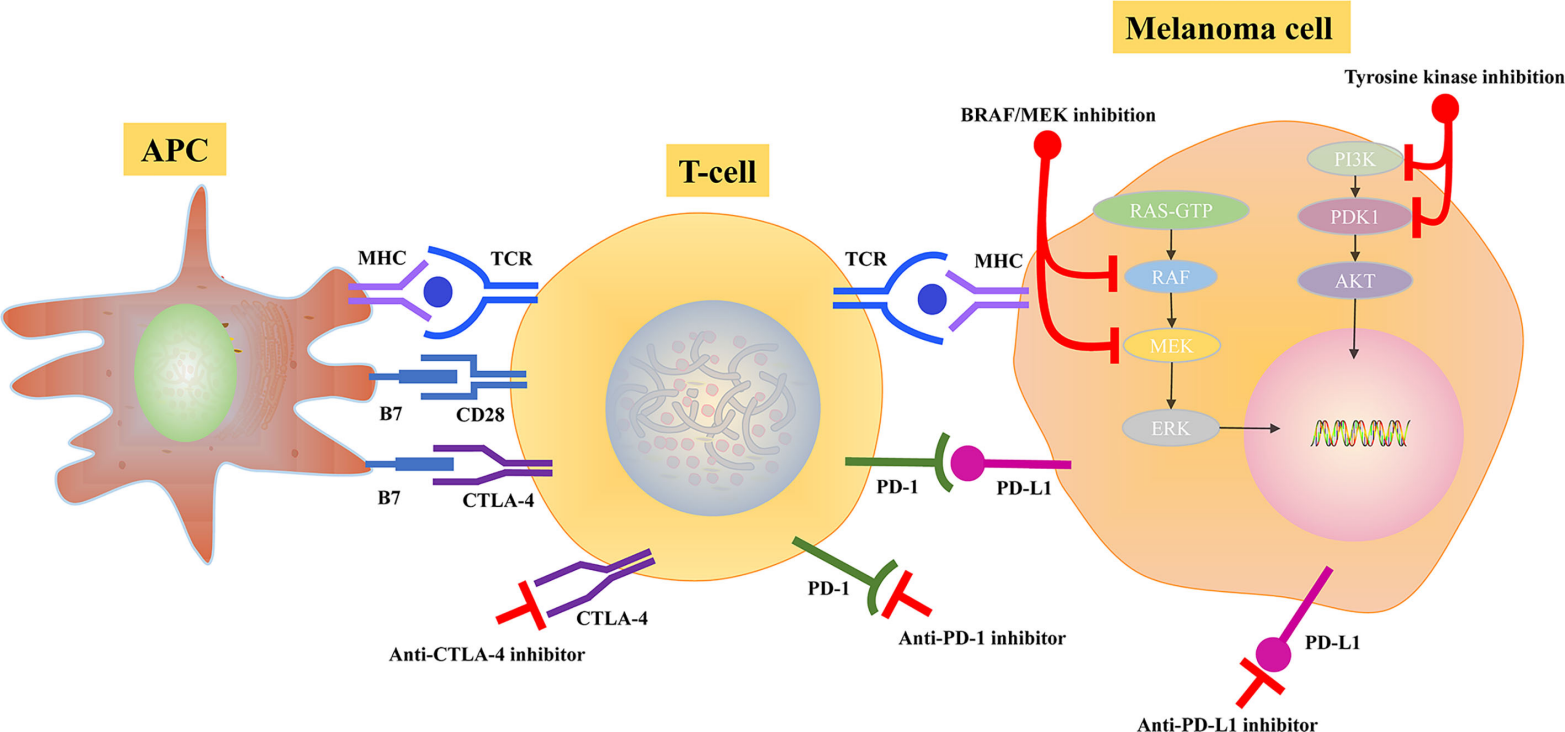
Potential mechanisms of BRAF/MEK inhibitor and immune checkpoint inhibition in melanoma.

Metastatic melanoma can benefit from immunotherapy, which can regulate and activate the immune system against cancer. The presence of lymphocytic infiltration is a hallmark of primary melanoma. The cytotoxic antigen 4 (CTLA-4) and the programmed death 1 (PD-1) immune checkpoints are the negative T-cell immune function modulators (**Table 2**). Inhibiting CTLA-4 and PD-1/PD-L1, leading to enhanced immune system activation, has contributed to the development of novel melanoma immunotherapies. During the interaction between antigen-presenting cells and T cells, CTLA-4, which functioned as a negative T-cell activation modulator in lymphoid tissues, is up-regulated on T cells due to the binding of tumor antigens to T-cell receptors. Modulating PD-L1 expression can reduce immune monitoring in the tumor microenvironment and prevent tumor immune escape by inhibiting T cell activation, implying that PD-L1/PD-1 signaling pathway is one of the main pathways of tumor immune escape. Approximately 30% of patients with metastatic melanoma who received anti-PD-1 therapy achieve long-term disease control. However, about two-thirds of individuals suffer from resistance and require further treatment (**Figure 3**).

A pooled analysis including KEYNOTE-001, KEYNOTE-002, and KEYNOTE-006 study endorses the use of pembrolizumab for treatment of advanced melanoma independent of BRAF V600 mutation status or previous treatment with BRAF inhibitor with or without MEK inhibitor. For patients with BRAF wild-type and BRAF V600-mutant melanoma, the objective response rate was 39.8% (447/1124) and 34.3% (149/434), 4-year progression-free survival rate was 22.9% (257/1124) and 19.8% (86/434), and 4-year overall survival was 37.5% (421/1124) and 35.1% (152/434), respectively. For patients with BRAF V600-mutant melanoma previously treated with or without BRAF inhibitor in combination with or without MEK inhibitor, the objective response rate was 28.4% (77/271) and 44.2% (72/163), 4-year progression-free survival rate was 15.2% (41/271) and 27.8% (45/163), and 4-year overall survival was 26.9% (73/271) and 49.3% (80/163), respectively (47). In the KEYNOTE-002 (NCT01704287) trial, pembrolizumab had an improvement in OS in patients with advanced melanoma compared with chemotherapy, but this was not statistically significant. The median OS was 13.4 months in the pembrolizumab 2mg/kg arm, 14.7 months in the pembrolizumab 10 mg/kg arm, and 11.0 months in the chemotherapy arm, respectively. The two-year survival rates for the pembrolizumab 2mg/kg, pembrolizumab 10 mg/kg, and chemotherapy groups were 36%, 38%, and 30%, respectively. Grade 3 or 4 treatment-related AEs occurred in 13.5% (N=178) of the patients who received pembrolizumab 2 mg/kg, 16.8% (N=179) of the patients who received pembrolizumab 10 mg/kg, and 26.3% (N=171) of the patients who received chemotherapy (48). In the KEYNOTE-006 study, the pembrolizumab groups had a median overall survival of 32.7 months, whereas the ipilimumab group had a median overall survival of 15.9 months after a median follow-up of 57.7 months. In addition, the pembrolizumab groups had a median progression-free survival of 8.4 months compared to 3.4 months in the ipilimumab group. Grade 3 or 4 treatment-related AEs occurred in 17% (N=555) of patients who received pembrolizumab, and 20% (N=256) of patients who received ipilimumab. Colitis (2% in the pembrolizumab groups and 6% in the ipilimumab group), diarrhoea (2% in the pembrolizumab groups and 3% in the ipilimumab group), and fatigue (<1% in the pembrolizumab groups and 1% in the ipilimumab group) were the most common grade 3 or 4 treatment-related AEs. 14% (N=536) of patients in the pembrolizumab arm and 18% (N=250) of patients in the ipilimumab group experienced any grade serious treatment-related AEs. In the pembrolizumab arm, one patient died from treatment-related sepsis. After nearly 5 years of follow-up, pembrolizumab demonstrated superiority over ipilimumab, supporting its usage in patients with advanced melanoma (49).

The CheckMate 037 study (NCT01721746) revealed that advanced melanoma patients who received nivolumab had more and higher durable responses than those who received chemotherapy. Median OS was 16 months versus 14 months. Median progression-free survival was 3.1 months in the nivolumab group and 3.7 months in the chemotherapy group. The overall response rate was 27% and 10% in the nivolumab and chemotherapy groups, respectively. The median response duration was 32 months in the nivolumab arm and 13 months in the chemotherapy group. Fewer treatment-related adverse events (AEs) (incidence ≥ 5%) were found in patients on nivolumab (77% *vs*. 82%). Grade 3 or 4 AEs occurred in 14% and 34% of patients in the nivolumab and chemotherapy groups, respectively. The most common AEs (incidence ≥ 10%) in patients receiving nivolumab were fatigue, pruritus, diarrhea, rash, nausea, and vitiligo (50). In the CheckMate 066 trial, the 5-year overall survival rate was 39% with nivolumab, 17% with dacarbazine, and 38% with dacarbazine and subsequent therapy, including nivolumab in patients with wild-type BRAF advanced melanoma. The 5-year analysis confirmed that nivolumab had a significant benefit over dacarbazine and extended long-term survival (51).

After a median follow-up of 22·1 months, the objective response rate of ipilimumab plus anti-PD-1 (pembrolizumab or nivolumab) and ipilimumab monotherapy was 31% (60/193) and 13% (21/162) for patients with advanced melanoma who had previously failed anti–PD-1 or anti–PD-L1 monotherapy. The overall survival of ipilimumab plus anti-PD-1 and ipilimumab monotherapy was 20.4 months and 8.8 months (p<0·0001), respectively. Besides, the progression-free survival was longer in the ipilimumab plus anti-PD-1 group than ipilimumab group (3.0 *vs*. 2.6 months; p=0·0019). Grade 3-5 AEs occurred in 31% (N=193) of the patients in the ipilimumab plus anti-PD-1 inhibitor group compared with 33% (N=162) in the ipilimumab alone group. The most common 3–5 AEs in the ipilimumab plus anti-PD-1 inhibitor group were diarrhoea or colitis (12%), and increased alanine aminotransferase or aspartate aminotransferase (12%). In the ipilimumab alone group, the most common 3–5 AEs were diarrhoea or colitis (20%), and increased alanine aminotransferase or aspartate aminotransferase (9%). The result indicated that ipilimumab combined with anti-PD-1 was superior to ipilimumab monotherapy as second-line immunotherapy for patients with advanced melanoma (52). The CheckMate 067 study exhibited that the median overall survival was dramatically improved with nivolumab plus ipilimumab compared to nivolumab or ipilimumab (over 60.0 *vs*. 36.9 *vs*. 19.9 months) after a minimum follow-up of 5 years in advanced melanoma. Besides, advanced melanoma patients treated with nivolumab plus ipilimumab had a 5-year overall survival rate of 52%, compared with 44% for nivolumab and only 26% for ipilimumab. The 5-year overall survival rates of patients with BRAF mutation treated with nivolumab plus ipilimumab, nivolumab, and ipilimumab were 60%, 46%, and 30%, respectively. The 5-year overall survival rates of patients without BRAF mutation treated with nivolumab plus ipilimumab, nivolumab, and ipilimumab were 48%, 43%, and 25%, respectively. Grade 3 or 4 treatment-related AEs occurred in 59% of patients who received nivolumab plus ipilimumab, 23% of patients who received nivolumab, and 28% of patients who received ipilimumab. Neither nivolumab alone nor nivolumab plus ipilimumab exhibited a significant decline in health-related quality of life. The findings showed that nivolumab plus ipilimumab or nivolumab alone might help patients with advanced melanoma achieve longer overall survival than ipilimumab (53). The response rates of anti-PD-1 inhibitors to patients with melanoma was 30-40%. When immune checkpoint therapy is combined with anti-PD-1 and anti-CTLA-4 inhibitors, the rate of innate resistance is reduced from 60-70% to 40-50%. Anti-PD-1/L1 before MAPK inhibitor treatment extends the duration of tumor remission. Prior immune checkpoint therapies are linked to prolonged progression-free survival in melanoma patients treated with MAPK inhibitor. Targeting M2-like tumor-associated macrophages (TAMs) and enhancing the clonal expansion of tumor-specific CD8+T cells may enhance anti-PD-L1 efficacy. In addition, prioritizing treatment with anti-PD1/L1 plus anti-CTLA-4 before MAPK inhibitor combination limits melanoma brain metastases (54).

In the COMBI-i trial, the objective response rate of combined spartalizumab (anti-PD-1 inhibitor), dabrafenib, and trametinib was 78% in advanced BRAF-mutant melanoma, and 44% of patients achieved a complete response. All patients experienced treatment-related AEs, with 72% suffering grade 3 or higher treatment-related AEs. The most common (≥8%) grade 3 or higher AEs were pyrexia, increased lipase, increased gamma-glutamyltransferase, and neutropenia increased blood creatine phosphokinase. 17% (N=35) of patients were discontinued due to treatment-related AEs, including immune-mediated hepatitis, paresthesia, hypokalemia, interstitial lung disease, increased alanine and aspartate aminotransferases, increased gamma-glutamyltransferase and generalized exfoliative dermatitis (55). The IMspire150 study exhibited that after a median follow-up of 18.9 months, progression-free survival was substantially longer in the atezolizumab group (vemurafenib, cobimetinib, and atezolizumab) than in the placebo group (vemurafenib, cobimetinib, and atezolizumab placebo) (15·1 *vs*. 10·6 months). The most common treatment-related AEs (incidence >30%) in patients receiving atezolizumab plus vemurafenib and cobimetinib are blood creatinine phosphokinase increased (51·3%), diarrhoea (42·2%), rash (40·9%), arthralgia (39·1%), pyrexia (38·7%), alanine aminotransferase increased (33·9%), and lipase increased (32·2%). The most common treatment-related AEs (incidence >30%) to atezolizumab placebo plus vemurafenib and cobimetinib are blood creatinine phosphokinase increased (44·8%), diarrhoea (46·6%), rash (40·9%), arthralgia (28·1%), pyrexia (26·0%), alanine aminotransferase increased (22·8%), and lipase increased (27·4%). In the atezolizumab group, 13% of patients and in the control group, 16% of patients discontinued therapy due to AEs. The study (IMspire150) revealed that atezolizumab was safe and tolerable when combined with vemurafenib and cobimetinib, and substantially improved progression-free survival in patients with BRAF V600 mutation-positive advanced melanoma (56).

The PIVOT-02 study (NCT02983045) exhibited the safety and efficacy of bempegaldesleukin, a CD122-preferential interleukin-2 pathway agonist, plus nivolumab in the first-line treatment of metastatic melanoma. After a median follow-up of 29.0 months, the objective response rate was 52.6% (N=38), and the complete response rate was 34.2% (N=38). Besides, the median progression-free survival of metastatic melanoma was 30.9 months. Bempegaldesleukin plus nivolumab caused grade 3 or 4 treatment-related AEs in 17.1% (N=41) of patients, which is consistent with anti-PD-1 inhibitors (16-17%) in this setting and substantially lower than nivolumab plus ipilimumab (55%) and BRAF plus MEK inhibitors (54%-68%) (57).

The EORTC 1325-MG/KEYNOTE-054 trial endorsed the use of adjuvant pembrolizumab treatment in patients with high-risk stage III melanoma. The adjuvant pembrolizumab group had a better 3.5-year distant metastasis-free survival rate after a median follow-up of 42.3 months compared with the placebo group in the intention-to-treat population (65.3% *vs*. 49.4%) (58). Besides, the adjuvant pembrolizumab group had a longer recurrence-free survival compared with the placebo group in the intention-to-treat population (Hazard ratio [HR], 0.56; 98.4% CI, 0.43 to 0.74). In addition, the occurrence of immune-related adverse events was linked to prolonged recurrence-free survival in the pembrolizumab group (59). In the KEYNOTE-716 (NCT03553836) trial, patients with resected high-risk stage II melanoma who received pembrolizumab in the adjuvant setting had a 35% reduction in disease recurrence or mortality compared to placebo (60). Patients with high-risk stage III melanoma treated with pembrolizumab had a 3-year recurrence-free survival rate of 63.7%, compared with 44.1% for placebo (61). For patients with resected high-risk melanoma, adjuvant ipilimumab therapy (3 mg/kg) had a significant difference in overall survival compared with high-dose interferon alfa-2b therapy (HR, 0.78; 95.6% CI, 0.61 to 0.99; P = 0.044) in the E1609 trial (62). In the CheckMate 238 trial, adjuvant nivolumab showed a prolonged recurrence-free survival benefit compared with ipilimumab in resected stage IIIB-C or IV melanoma at a minimum follow-up of 4 years (51.7% *vs*. 41.2%, P=0.0003) (63). The IMMUNED trial compared the effectiveness of adjuvant nivolumab plus ipilimumab or nivolumab monotherapy to placebo in patients with resected stage IV melanoma. The median recurrence-free survival was not reached in the nivolumab plus ipilimumab arm, but the median recurrence-free survival in the nivolumab arm was 12.4 months and 6.4 months in the placebo arm after a median follow-up of 28.4 months (64). The OpACIN and OpACIN-neo trials exhibited that neoadjuvant ipilimumab plus nivolumab had high pathologic response rates in patients with macroscopic stage III melanoma. None of the patients in the OpACIN study with a pathologic response (7/9) had relapsed after a median follow-up of 4 years. Additionally, 2-year relapse-free survival was 84% for all patients, 97% for those who achieved a pathological response, and 36% for those who did not in the Opacin-neo study (65). In a pooled analysis, neoadjuvant therapy in melanoma with ipilimumab plus nivolumab, anti-PD-1 inhibitor, and targeted inhibitor showed pathological complete response rates of 43%, 20%, 47%, respectively (66).

## Adoptive Cell Transfer (ACT)

Adoptive cell transfer (ACT) utilizing tumor-infiltrating lymphocytes or modified T cells has shown encouraging outcomes in patients with melanoma (67). Patients with metastatic melanoma who have progressed after receiving immune checkpoint inhibitors or targeted therapies have limited therapeutic alternatives. Tumor-infiltrating lymphocyte-administered adoptive cell therapy has shown to be effective in treating metastatic melanoma. Lifileuel is an autologous tumor-infiltrating lymphocyte (TIL) product that has shown sustained responses in patients with metastatic melanoma who have progressed following immune checkpoint inhibitors or targeted therapies. The ORR to lifileuel was 36% (N=66) for metastatic melanoma, consisting of two complete responses and 22 partial responses, and the disease control rate reached 80% (68). In a clinical phase I/II study (NCT03296137), tumor-infiltrating lymphocytes (TILs)-based adoptive cell therapy (ACT) with immune checkpoint inhibitors was assessed across various solid cancer types. Five patients, including two partial responses in patients with cholangiocarcinoma and head-and-neck cancer, experienced significant tumor regressions of 30%–63%. Moreover, clinical effectiveness is related to the phenotypic characterization of rapid expansion protocol (REP) TILs, particularly alpha-integrin CD103 expression and tumor mutational burden (TMB). Additionally, the potential of employing immune checkpoint inhibitors to facilitate TIL development and treatment also needs to be investigated (69). The study of chimeric antigen receptor (CAR) T-cell treatments is progressing, but the anticipated benefits in hematologic malignancies have yet to be shown in solid tumors. The paucity of CAR-T cells that migrate from blood vessels to the target site, the immunosuppressive tumor microenvironment inside the malignant tumor, and the appropriate identification of target antigen to prevent on-target/off-tumor toxicities are all significant obstacles (70).

## Cytokines (Interleukin-2)

Interleukin-2 (IL-2) is an important cytokine with a variety of effects on the immune system. It has extensive effects on the development and expansion of T cell subsets, particularly CD8+ T cells, and has anti-tumor activity in advanced melanoma. In 1998, the Food and Drug Administration (FDA) approved high-dose IL-2 for the treatment of patients with metastatic melanoma. In a phase II trial in 1994, 7% (N=129) of patients with metastatic melanoma experienced complete regression of disease and 10% (N=140) of patients experienced partial regression (71). In a retrospective study, the overall objective response rate in patients with metastatic melanoma treated with IL-2 was 16% (N=270), with 17 complete responses and 26 partial responses (72). In another retrospective study, high-dose IL-2 treatment maintained antitumor efficacy in patients with metastatic melanoma who had progressed after receiving PD-1 and PD-L1 suppression. The best overall response rate (ORR) to high-dose IL-2 was 22.5% for metastatic melanoma, with four complete responses and five partial responses. These findings encourage further investigation of high-dose IL-2 as an immunotherapy for metastatic melanoma patients. Moreover, the combination of immune checkpoint inhibitors and IL-2 therapy deserves further exploration. In addition, the exploration of targeting IL-2 is a critical complement to current immunotherapy and may help us better understand immunotherapy signal transduction (73).

## T-Cell Agonists Targeting 4-1BB/OX40

Immune checkpoint inhibitors can block cancer cells from escaping immune surveillance and improve T cell antitumor efficacy. Besides, antitumor T-cell functions are enhanced by targeting costimulatory molecules expressed on T-cell surfaces such as 4-1BB, OX40, inducible T-cell costimulator, and glucocorticoid-induced TNF receptor (GITR). 4-1BB (CD137) belonging to the TNF receptor family is expressed on a broad range of cell types, including activated T cells, dendritic cells (DCs), NK cells, B cells, monocytes, and neutrophils, and its activation caused by antibody ligation or 4-1 BB ligand contributes to T-cell activation (74). Utomilumab and urelumab, which are monoclonal antibody agonists (mAb-AGs) targeting 4-1BB, have shown strong anti-tumor efficacy in preclinical models, but clinical studies have been limited by only marginal efficacy or serious liver toxicity. According to data from the trials (NCT00309023, NCT00612664, NCT01471210), urelumab monotherapy (0.1 mg/kg every 3 weeks) was well tolerated and immunoreactive, indicating that urelumab monotherapy and in combination with other immuno-oncology agents can be further assessed in patients with advanced solid tumors and lymphoma (75). Additionally, LVGN6051 is a novel 4-1BB mAb-AG, which improves the antitumor efficacy *via* regulating agonistic strength and FcγR affinity without liver damage (76).

The preclinical and clinical data indicated that melanoma treatment may move forward using agonists like OX40 (CD134, TNFRSF4), CD137, CD40, GITR, and CD27 activating co-stimulatory pathways (77). OX40 is a potent costimulatory protein in the tumor necrosis factor receptor superfamily, which actively participates in the positive regulation of CD4+ and CD8+ T cell activity and inhibits the activity of regulatory T cells, showing an effective anti-tumor effect. In a phase I trial (NCT02315066), the safety and tolerability of ivuxolimab (an OX40 agonist monoclonal antibody) were assessed in patients with locally advanced or metastatic cancers. A partial response was obtained in three patients (N=52), and 56% of patients gained disease control. At 0.1 to 3.0 mg/kg, elevated CD4+ central memory T-cell proliferation and activation, as well as clonal expansion of CD4+ and CD8+ T cells, were seen in peripheral blood. An elevated OX40 expression and immune cell infiltration were observed in on-treatment tumor samples (78).

## Potential Mechanisms of Immunotherapy Resistance

Even though checkpoint inhibitors have enhanced long-term survival, roughly 40%-50% of tumors are unresponsive to single-agent immune checkpoint inhibition, and these patients eventually develop drug resistance. Tumor-intrinsic resistance mechanisms to immune checkpoint inhibitor therapy included PD-L1 expression, mutational burden, neoantigen expression, epigenetic variations, type II interferon signaling, and antigen presentation pathways. In addition, tumor-extrinsic resistance mechanisms to immune checkpoint inhibitor therapy mainly included microbiome, PD-L1 expression on immune cells, tumoral and peripheral immune, and cell composition (79).

In the KEYNOTE-006 study, TMB, GEP, and PD-L1 were significantly associated with best overall response (BOR), progression-free survival (PFS), and overall survival (OS) in the pembrolizumab group. Patients with high TMB (≥175) and GEP (≥-0.318) levels had a higher response rate to pembrolizumab treatment than those with low TMB and GEP levels (54% *vs*. 14%). Besides, patients with high TMB (≥175) and PD-L1 MEL scores (≥2) showed higher response rates to pembrolizumab treatment than those with low TMB and PD-L1 MEL scores (51% *vs*. 33%). However, only GEP exhibited substantial associations with PFS and OS in the ipilimumab group (80).

Patients with high TMB or high inflammatory signature score had a longer PFS and OS across all three treatment groups (nivolumab plus ipilimumab group, nivolumab group, and ipilimumab group) in the CheckMate 067 (NCT01844505) study. In the CheckMate 066 (NCT01721772) study, high TMB were significantly associated with PFS (HR 0.33; 95% CI: 0.16, 0.69; p=0.0031) and OS (HR 0.43; 95% CI: 0.20, 0.91; p=0.027) in patients treated with nivolumab. Based on the results of the CheckMate 066 and CheckMate 067 studies, patients with high TMB and no BRAF mutation had longer survival. Besides, weak associations between PD-L1, TMB, and the inflammatory signature were found. A combination of TMB, inflammatory gene expression signature, and BRAF mutation status may predict response to immune checkpoint blockade in advanced melanoma (81, 82).

In comparison to patients with PFS >12 months, those with early PFS events (≤12 months) had lower expression levels of known genes and gene expression signatures (GESs) associated with immune infiltration, such as interferon (IFN)-γ and T cell–inflamed in the COMBI-i trial. Lower tumor mutational burden (TMB) or T cell–inflamed GES levels, higher specific immunosuppressive TME signatures levels, and increased baseline circulating tumor DNA (ctDNA) levels were strongly associated with early PFS events. Biomarker profiles exhibited that patients with complete response (CR) had relatively low or undetectable baseline ctDNA levels. In subgroup analysis, the commonly used immunotherapy response biomarkers TMB and T cell–inflamed GES were not associated with CR, but patients who achieved CR had lower baseline levels of immunosuppressive TME signatures. When comparing baseline and 2 to 3 weeks of treatment, a rise in T cell-inflamed GES was found independent of subsequent patient progression, while MAPK pathway activity scores (MPAS) and cell cycle GESs reduced from baseline to 2 to 3 weeks of treatment. Biopsy analysis of patients with early PFS events at 8- to 12-week biopsies exhibited a reduction in T-cell inflammatory GES and an increase in MPAS. Correlative analysis between GES levels and tumor shrinkage following treatment with spartalizumab plus dabrafenib and trametinib exhibited that PI3K pathway GES levels were correlated with best overall tumor reduction, indicating that a compensatory signaling pathway for MAPK inhibition may be responsible for the lack of early response (55).

In the PIVOT-02 study (NCT02983045) study, the improved objective response rate and progression-free survival were observed in baseline tumor biopsies with high IFN-γ GEP, high CD8+ TIL, high CD74, and high HLA-E. After treatment, CD4+ T cells were significantly upregulated, NK cell polyfunctionality was reduced, and polyfunctional CD8+ T cells were significantly enhanced only in patients with an objective response. The elevated polyfunctional response in CD8+ and CD4+ T cells seems to be promoted by the generation of cytokines with effector functions (57).

The mechanism of immunotherapy resistance mainly includes immune-desert or immune-cold tumor immunophenotypic models. In the immune-cold phenotype, stromal or vascular factors are responsible for the periphery of immune cells in the tumor microenvironment, leading to resistance to immunotherapy. In the immune-desert phenotype, the evidence of immune infiltrates in the tumor microenvironment is absent. In the immune-desert or immune-cold tumor, the number of regulatory cells entering the tumor microenvironment elevates, the secretion of immunosuppressive cytokines such as IL-8, TGF-β, VEGF elevate, and the expression of inhibitory receptors such as PD-1, CTLA-4, TIM3, LAG3, VISTA, BTLA, CD160 on T cells is enhanced. Combination therapy strategies that include immune checkpoint inhibitors may make the tumor microenvironment more immune-infiltrated, thus improving the effectiveness of immune checkpoint inhibitors (83). Melanoma cells with JAK1/2 deletion are resistant to IFN-induced anticancer activity, whereas melanoma cells with B2M loss block melanoma cells from being recognized by antigen-specific T cells and maybe therefore resistant to cytotoxicity. *In vivo*, intratumoral administration of Toll-like receptor 9 agonists and anti-PD-1 inhibitors can activate innate and adaptive immunity *via* natural killer (NK) and CD8 T cells, addressing JAK1/2 loss of resistance. The activation of NK-cell and CD4 T-cell using CD122-preferential interleukin (IL)-2 pathway agonists can address B2M-loss resistance. Stimulating NK cells and activating IFN signaling *via* pattern recognition receptors address the PD-1 inhibitor resistance caused by JAK1/2, and B2M pathogenic mutations mediated by defective IFN receptor and antigen presentation pathways (84).

## Conclusions and Future Perspectives

Pharmaceutical advancements have opened the path for precision cancer treatment in the present era of melanoma management. With the introduction of innovative therapeutic methods, such as BRAF and MEK inhibitors and immune checkpoint inhibitors, the prognosis of metastatic melanoma patients has dramatically enhanced in recent years. The optimal treatment strategy and medication sequence for patients with BRAF mutation or BRAF wild-type melanoma are still unclear. Most patients will only receive partial and short-term benefits from systemic therapy, and drug resistance and disease progression will almost inevitably occur. Combination therapies for different resistance mechanisms are a promising approach, but less toxic drug strategies are critical. Given the survival advantages in metastatic melanoma and the outcomes of the early-stage clinical trials, these treatment strategies can be used as adjuvant treatments for high-risk resected stage III melanoma. For neoadjuvant therapy of high-risk stage III melanoma, these treatment strategies have also been explored in several studies, and preliminary data indicates that they might be a very promising strategy in this scenario. The current results still need to be confirmed by prospective clinical trials before they can be used in daily clinical practice. Ongoing clinical research can improve our understanding of melanoma, develop more effective and less toxic treatments, and improve the prognosis of patients with melanoma. Although immune checkpoint inhibitors have revolutionized melanoma patients, successful treatments for melanoma are still a long way off. 

## Author Contributions

HHZ, FL, HRZ, and CZ designed the study and supervised it. HHZ and FL collected data. HHZ performed statistical analysis. HRZ interpreted data and drafted the manuscript. CZ contributed to administrative, technical and material support. All authors contributed to the article and approved the submitted version.

## Funding

This present study was supported in part by the National Natural Science Foundation of China (grant numbers: 81660755), and the Science and Technology Project of Shenzhen of China (grant numbers: JCYJ20190808162605484).

## Conflict of Interest

The authors declare that the research was conducted in the absence of any commercial or financial relationships that could be construed as a potential conflict of interest.

## Publisher’s Note

All claims expressed in this article are solely those of the authors and do not necessarily represent those of their affiliated organizations, or those of the publisher, the editors and the reviewers. Any product that may be evaluated in this article, or claim that may be made by its manufacturer, is not guaranteed or endorsed by the publisher.
